# Tracheid and Pit Dimensions Hardly Vary in the Xylem of *Pinus sylvestris* Under Contrasting Growing Conditions

**DOI:** 10.3389/fpls.2021.786593

**Published:** 2021-12-21

**Authors:** Magdalena Held, Andrea Ganthaler, Anna Lintunen, Walter Oberhuber, Stefan Mayr

**Affiliations:** ^1^Department of Botany, University of Innsbruck, Innsbruck, Austria; ^2^Institute for Atmospheric and Earth System Research (INAR)/Forest Sciences, University of Helsinki, Helsinki, Finland

**Keywords:** xylem anatomy, water transport, interconduit pit, *Pinus sylvestris*, tracheid, plasticity

## Abstract

Maintaining sufficient water transport *via* the xylem is crucial for tree survival under variable environmental conditions. Both efficiency and safety of the water transport are based on the anatomical structure of conduits and their connections, the pits. Yet, the plasticity of the xylem anatomy, particularly that of the pit structures, remains unclear. Also, trees adjust conduit dimensions to the water transport distance (i.e., tree size), but knowledge on respective adjustments in pit dimensions is scarce. We compared tracheid traits [mean tracheid diameter *d*, mean hydraulic diameter *d*_*h*_, cell wall reinforcement (*t*/*b*)^2^], pit dimensions (diameters of pit aperture *D*_*a*_, torus *D*_*t*_, margo *D*_*m*_, and pit border *D*_*p*_), and pit functional properties (margo flexibility *F*, absolute overlap *O*_*a*_, torus overlap *O*, and valve effect *V*_*ef*_) of two Scots pine (*Pinus sylvestris* L.) stands of similar tree heights but contrasting growth rates. Furthermore, we analyzed the trends of these xylem anatomical parameters across tree rings. Tracheid traits and pit dimensions were similar on both sites, whereas *O*_*a*_, *O*, and *F* were higher at the site with a lower growth rate. On the lower growth rate site, *d*_*h*_ and pit dimensions increased across tree rings from pith to bark, and in trees from both sites, *d*_*h*_ scaled with pit dimensions. Adjusted pit functional properties indicate slightly higher hydraulic safety in trees with a lower growth rate, although a lack of major differences in measured traits indicated overall low plasticity of the tracheid and pit architecture. Mean hydraulic diameter and pit dimension are well coordinated to increase the hydraulic efficiency toward the outer tree rings and thus with increasing tree height. Our results contribute to a better understanding of tree hydraulics under variable environmental conditions.

## Introduction

To cope with variable water availability, trees must maintain sufficient water transport (McDowell et al., 2008; Adams et al., 2017). Water transport is, besides mechanical support and storage, the main function of the xylem and is based on the xylem’s anatomical structures (Kramer and Kozlowski, 1960; Zimmermann, 1983). Xylem conduits transport water from roots to leaves based on a water potential gradient (negative pressure or tension) induced by transpiration from the stomata (Dixon and Joly, 1895). Water flow is restricted by resistances along the entire pathway and the cumulative resistance thus increases with pathway length (Ryan and Yoder, 1997). Resistance of single xylem elements decreases approximately with the fourth power of conduit lumen diameter (Hagen-Poiseuille’s law; Zimmermann, 1983), and trees counteract pathway length resistance by, respectively, widening their conduits with distance to the treetop (or branch tip; Anfodillo et al., 2013; Carrer et al., 2015; Lazzarin et al., 2016; Olson et al., 2021). However, with increasing conduit lumen diameter, the risk of hydraulic failure can increase (e.g., Rosner et al., 2016).

Larger conduits may have a higher probability of vulnerable pits (interconduit connections) as more pits are embedded in their cell walls (Hargrave et al., 1994; Jacobsen et al., 2019). Bordered pits are crucial structures regarding both hydraulic efficiency (by representing the main resistance within the xylem; Domec et al., 2006) and hydraulic safety (i.e., resistance against hydraulic failure; Zimmermann, 1983). Both water flow between conduits and the probability of air seeding, which may cause embolism and block water transport, increase with a pore size of pit membranes (e.g., Zimmermann, 1983; Tyree et al., 1994; Hacke and Sperry, 2001; Choat et al., 2008). In conifers, valve-like structured pits counteract this trade-off: their pit membrane, formed by the primary wall, consists of an outer microfibril web (margo), and a central thickening (torus). The secondary cell wall protrudes to form a border with an aperture on each side of the membrane (toward both adjacent tracheids). If neighboring conduits are functional, the membrane is in a relaxed, central position, exposing both margo pores and pit apertures to water flow. If one conduit is air filled (i.e., embolized; Zimmermann, 1983), the pressure difference between the adjacent conduits causes the torus to seal the pit aperture, covering large margo pores to prevent air seeding (Bailey, 1916; Zimmermann, 1983; Sperry and Tyree, 1990). However, under critically high tensions pits may fail. Several air-seeding mechanisms have been proposed for this process (Sperry and Tyree, 1990; Domec and Gartner, 2002; Hacke et al., 2004; Cochard, 2006; Cochard et al., 2009; Delzon et al., 2010), which imply that dimensions of the pit border, margo, torus, and aperture, and particularly the ratios of these dimensions play a crucial role concerning the resistance against air seeding (e.g., Cochard, 2006; Hacke and Jansen, 2009; Delzon et al., 2010; Jansen et al., 2012; Schulte and Hacke, 2021). Hacke et al. (2004) found both smaller margo and pit aperture to respond to higher resistance to embolism. Furthermore, Hacke and Jansen (2009) reported that the ratio of torus and pit membrane area to pit aperture increase with resistance to embolism. According to Schulte et al. (2015), a high total area of margo pores and pit diameter reduce the hydraulic resistance of pits, while margo pore size and number also influence the hydraulic efficiency (Hacke et al., 2004). Thus, an optimized pit architecture and torus–margo ratio is important for hydraulics of length-limited tracheids in conifers (Hacke et al., 2004). The potential plasticity of xylem anatomical structures may be relevant for tree survival and is the subject of several studies. For example, Castagneri et al. (2015) found temperature and precipitation to influence conduit dimensions in *Picea abies* (L.) Karst. In contrast, Olson et al. (2018, 2021) suggested that trees hardly adjust their conduit dimensions to environmental conditions and that different conduit dimensions at the commonly sampled breast height can occur as a secondary effect due to different tree heights (or branch lengths). Furthermore, knowledge concerning the potential plasticity in conifer pit dimensions and the coordination of pit and conduit dimensions remains poor (e.g., Lazzarin et al., 2016; Losso et al., 2018).

In our study, we analyzed xylem anatomical traits in *Pinus sylvestris* L. sampled on two sites with contrasting growth rates but similar tree heights (tree-ring width 727 ± 15 μm *vs.* 2,724 ± 135 μm; tree height 17.4 ± 0.4 m *vs.* 16.9 ± 0.3 m) to reveal if trees adjust these traits to growing conditions independently from the distance to the treetop. Measured tracheid traits were mean tracheid lumen area (*a*), mean tracheid lumen diameter (*d*), mean hydraulic diameter (*d*_*h*_), and cell wall reinforcement [(*t*/*b*)^2^; Hacke et al., 2001]. From the pits, we measured border diameter (*D*_*p*_), margo diameter (*D*_*m*_), torus diameter (*D*_*t*_), and aperture diameter (*D*_*a*_), with which we calculated the following functional properties according to Delzon et al. (2010) and Domec et al. (2008): margo flexibility (*F*), absolute overlap (*O*_*a*_), torus overlap (*O*), and valve effect (*V*_*ef*_). Our study aimed to (a) explore differences in these tracheid and pit traits between trees that are of similar height but have experienced different growing conditions, and (b) to better understand the within-tree variation and coordination of tracheid and pit characteristics. We hypothesized that (1) trees on the limited site have a higher hydraulic safety than trees on the favorable site thanks to adjusted tracheid and pit characteristics, particularly *D*_*m*_, *D*_*t*_, *D*_*a*_, and resulting functional properties, and (2) trees adjust their tracheid and pit characteristics according to the distance from the treetop. Thus, at breast height we expected anatomical adjustments increasing the hydraulic efficiency from the inner to the outer tree rings.

## Materials and Methods

### Site Description

This study was performed in the Eastern Alps, on two forest sites situated in the Inn valley west of Innsbruck (Tyrol, Austria). Tree growth was limited on one site, whereas the other site had more favorable conditions. The limited sampling site (Tschirgant; N47°13.922′, E10°50.886′) lies at 745 m asl. Its rock bed consists of post-glacial rockfall material (Patzelt and Poscher, 1993) classified as dolomite (Land Tirol, 2021) and has a low water-holding capacity due to shallow, stony soils, and low nutrient availability (Oberhuber et al., 2014). A weather station close to the site (Haiming) measured a mean annual temperature of 7.4°C and mean annual precipitation of 716.7 mm (ZAMG, 2021). *P. sylvestris* dominated this site with *P. abies* and *Larix decidua* Mill., occurring occasionally in the understory. The natural forest types in the sampled area are *Erico-Pinetum dorycnietosum germanici* (dominated by *P. sylvestris*) and *Carici albae-Tilietum cordatae typicum* (broad-leaf dominated), of which the latter has a higher water and nutrient demand (Land Tirol, 2021).

The favorable site (Mieming; N47°18.542′, E11°01.019′) lies at 825 m asl on a gravel terrace, which is partly covered by scree material (von Klebelsberg, 1935), which is siliceous but rich in carbonates (Land Tirol, 2021). As the favorable site lies about 10 km northeast of the limited site, the climatic conditions are similar; however, due to a precipitation gradient toward the west, the favorable site may experience a slightly higher mean annual precipitation. Here, *P. sylvestris* grew mixed with *P. abies*. The natural forest type in the sampled area is *Carici albae-Tilietum cordatae typicum* (Land Tirol, 2021). Sampled trees on the limited site were 17.4 ± 0.4 m high, with an average cambial age at breast height of 160 ± 7 years, whereas, trees on the favorable site were 16.9 ± 0.3 m high (difference between sites non-significant; *p* = 0.162), with a cambial age at breast height of 32 ± 1 years (mean ± SE).

### Sampling

On each site, we selected ten *P. sylvestris* trees of similar height and sampled them using a tree increment borer (5.15 mm diameter, 400 mm length; Haglöf, Mora 400). We extracted two cores per tree at breast height (1.25–1.35 m) from the south-facing side of the trunk in November 2017 (limited site) and March 2018 (favorable site) for tree-ring and anatomical analysis *via* light and scanning electron microscopy. We also recorded tree circumference at breast height and tree height, the latter using a laser rangefinder (TruPulse 200 Series, Laser Inc. Technology, Centennial, CO, United States).

### Tree-Ring Analysis

One core per tree was used for the tree-ring analysis. Using a measuring table (LINTAB 6, RINNTECH, Heidelberg, Germany) connected to the tree-ring program TSAPwin Scientific (ver. 4.80e RINNTECH), earlywood and latewood widths were measured (resolution 1 μm). The boundary between earlywood and latewood was set to a point where double cell wall thickness was equal or larger than the tracheid lumen area (both in radial direction). In *P. sylvestris*, the transition from earlywood to latewood is abrupt (Schweingruber, 1993), allowing for a relatively accurate determination of the boundary. Total tree-ring width was calculated as the sum of earlywood and latewood width. We checked the quality of cross-dating using the COFECHA software (Holmes, 1986).

### Xylem Anatomical Analysis

For tracheid analysis, we extracted selected tree rings (tree-ring samples) from the cores of five trees per site (randomly selected; previously used for tree-ring analysis). In tree cores from the limited site, we selected every 20th tree ring starting from the outermost tree ring (2017) to compare the measured tracheid traits between sites (to sample the whole life span with regular intervals as on the limited site). To analyze trends in tracheid traits from the inner to the outer tree rings, we used the same tree-ring samples as for the site comparison but added additional tree-ring samples to make the trends better visible. From one of the trees, we selected every 10th tree ring throughout the whole life span, and from the remaining trees, we selected every 10th tree ring within the innermost 50 tree rings (because trees grow usually faster while they are young; Baker, 1950). In tree cores from the favorable site, we selected every 10th tree ring for site comparison and trend analysis. Also, we compared the tracheid traits of the outermost tree ring separately, as this tree ring was formed when the trees on both sites reached similar heights. For pit analysis, we used the second tree core of all ten trees per site and selected tree rings according to the same scheme as for tracheid analysis (Supplementary Table 1).

For tracheid analysis, tree-ring samples were soaked in ethanol/glycerol/water solution (1:1:1, v/v/v) for at least 5 days (Losso et al., 2018), before cross-sections (around 30 μm thickness to avoid breaking and to get a good contrast) were produced using a sliding microtome (Sledge Microtome G.S.L. 1, Schenkung Dapples, Zurich, Switzerland). The sections were stained with Etzold’s solution for at least 15 min. Images were taken with a light microscope (Olympus BX41, Olympus Austria, Wien, Austria), connected to a digital camera (ProgRes CT3, Jenoptik, Jena, Germany) at 10 × magnification and with a resolution of 2,048 × 1,536 pixels (see Supplementary Figures 1A–F for an example tree ring from the limited and favorable site, respectively). With the ImageJ 1.45 software (National Institutes of Health, Bethesda, MD, United States), we measured the cross-sectional tracheid lumen area in both earlywood and latewood in at least five radial files, whereby we differentiated tracheid lumina from walls by applying a threshold value on gray-scale images and edited the images manually where necessary. From each image, we cropped the area of interest manually. In tree rings covering several images, we analyzed each image separately. The mean tracheid lumen area was calculated for each tree-ring sample. The lumen diameter was calculated for each tracheid assuming square-shaped lumina and then averaged (mean tracheid lumen diameter, *d*). Tracheid diameter distribution based on 2-μm classes was calculated for each site. Furthermore, the mean hydraulic diameter (*d*_*h*_), which weighs conduit diameters with respect to the theoretical hydraulic conductance (Sperry et al., 1994), was calculated according to Sperry and Hacke (2004) as Σ*d*^5^/Σ*d*^4^, where *d*_*t*_ is the diameter of each analyzed tracheid. For calculating cell wall reinforcement [(*t*/*b*)^2^] according to Hacke et al. (2001), tracheid lumen diameter (*b*) and wall thickness (*t*) were manually measured (therefore *b* ≠ *d*) on 10 selected tracheids per tree-ring sample with a hydraulic diameter of *d*_*h*_ ± 6 μm. The (*t*/*b*)^2^ is typically measured on tracheids within a certain diameter range around *d*_*h*_. The diameter range applied here was necessary for small tree rings to reach the sample number of 10.

**FIGURE 1:**
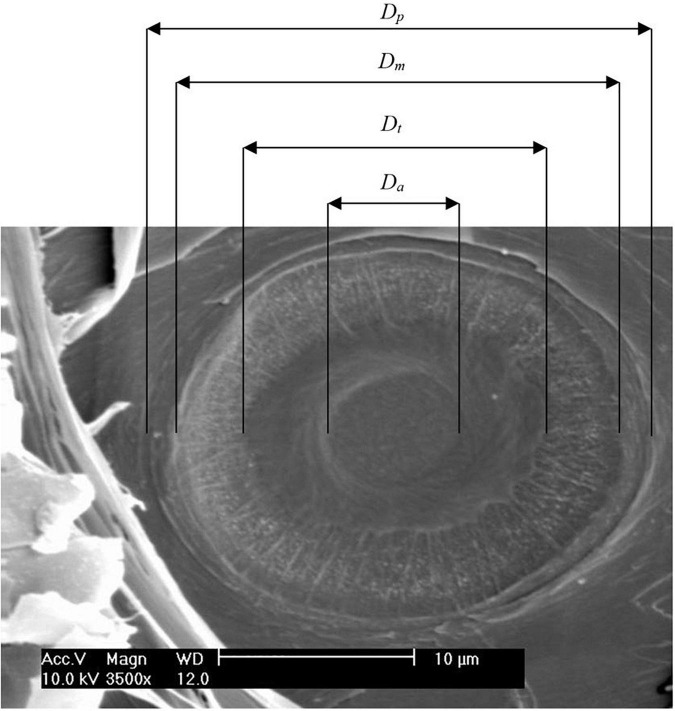
A dissected pit is viewed *via* a scanning electron microscope. Diameter of pit (*D*_*p*_), margo (*D*_*m*_), torus (*D*_*t*_), and pit aperture (*D*_*a*_) are indicated.

For pit analysis, tree-ring samples were soaked in 50% ethanol for at least one night. Using a sliding microtome (see tracheid analysis), the samples were radially cut to produce woodblocks of approximately 5 mm × 5 mm × 2 mm, which were dried at 70°C for at least one night. Next, they were fixed on object stages and dried again at 40°C overnight. Finally, they were sputtered with gold using Leica EM SCD050 (Leica Microsystem, Wetzlar, Germany). A scanning electron microscope (SEM model XL 20, Philips, Amsterdam, Netherlands) was used to measure the dimensions of ten pits per sample, which were randomly cut open by the microtome. The entire selected tree ring was searched for measurable pits (i.e., opened pit chamber, all pit structures visible, horizontal orientation). When the number of measurable pits was insufficient, pits of adjacent tree rings were included in the measurements. Most of the measurable pits were found in the earlywood (as the pits of larger tracheids are more likely to be cut open). Measured dimensions were the diameter of pit aperture (*D*_*a*_), torus (*D*_*t*_), margo (*D*_*m*_), and pit border (*D*_*p*_). From these dimensions (Figure 1), functional properties were calculated for each pit: the absolute torus overlap (*O*_*a*_ = *D*_*t*_/*D*_*a*_) was calculated according to Domec et al. (2008), and the margo flexibility [*F* = (*D*_*m*_ −*D*_*t*_)/*D*_*m*_], torus overlap [*O* = (*D*_*t*_ −*D*_*a*_)/*D*_*t*_], and valve effect (*V*_*ef*_ = *F* × *O*) were calculated according to Delzon et al. (2010).

### Data Analysis and Statistics

All values are given as mean ± standard error (SE). For statistical analyses, R (version 4.0.1, R Core Team, 2020) was used in RStudio (version 1.3.959, RStudio Team, 2020) by applying the packages tidyverse (version 1.3.0, Wickham et al., 2019) and Hmisc (version 4.4-1, Harrell and Dupont, 2020). The Shapiro–Wilk Normality tests were carried out before further analyses. To analyze differences between sites, parameters were averaged per tree and tested with Welch’s *t*-test if normally distributed, otherwise the Mann–Whitney *U* tests were carried out. For correlations, parameters were averaged per year and site. Correlations were tested using either the Pearson product–moment coefficient or Spearman’s rank correlation coefficient (in the case of non-normally distributed data).

## Results

### Radial Growth

Trees on the limited site were older than those on the favorable site. On average, tree cores from the limited site contained 160 ± 7 tree rings, whereas those from the favorable site contained 32 ± 1 (*p* < 0.001; all values given as mean ± SE). No false or missing rings were observed in any tree individual. However, the pith was missing in some cores, and thus, the cambial age at breast height was underestimated by approximately 3 years in some trees. Trees on the limited site had larger circumferences at breast height (93.0 ± 5.3 cm) than those on the favorable site (70.6 ± 3.7 cm; *p* = 0.003). The average earlywood width was 515 ± 12 μm *vs.* 2,099 ± 156 μm (*p* < 0.001) and latewood width was 212 ± 5 μm *vs.* 625 ± 36 μm (*p* < 0.001) on the limited *vs.* favorable sites, respectively, resulting in an average tree-ring width of 727 ± 15 μm on the limited and 2,724 ± 135 μm on the favorable site (*p* < 0.001). The ring width chronology of the limited site covered a time span (i.e., maximum number of tree rings) of 192 years. Tree-ring width and earlywood width exhibited a decreasing trend from pith to bark (*p* = 0.002 and *p* < 0.001), whereas latewood width remained constant. The ring width chronology of the favorable site covered a time span of 37 years. While tree-ring width and earlywood width exhibited a decreasing trend, an increasing trend in latewood width was observed (each *p* < 0.001; Supplementary Figure 2). Thus, the earlywood–latewood ratio decreased with cambial age on both sites.

### Tracheid Traits

**FIGURE 2:**
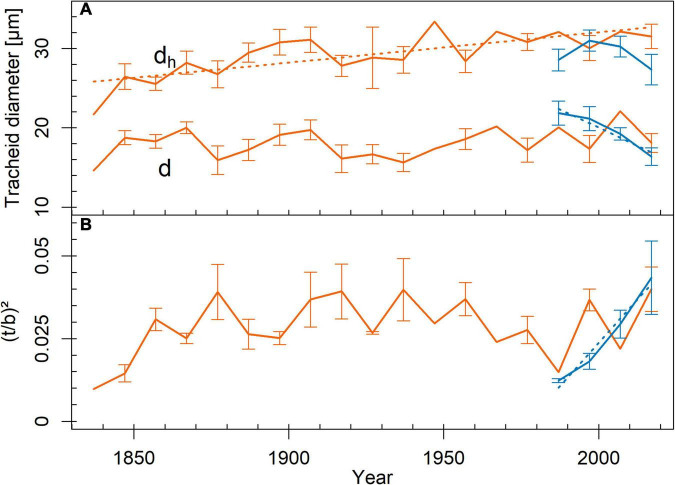
Trends in tracheid traits across tree rings from pith to bark (red = limited site, blue = favorable site). **(A)** mean tracheid diameter (*d*; *r*^2^_*favorable*_ = 0.94, *p* = 0.032) and mean hydraulic diameter (*d*_*h*_; *r*^2^_*limited*_ = 0.57, *p* < 0.001), **(B)** cell wall reinforcement [(*t*/*b*)^2^; *r*^2^_*favorable*_ = 0.97, *p* = 0.015]. Mean ± SE. Measurement points without SE bars represent tree rings, which were sampled only in one tree individual. Given *r*^2^- and *p*-values resulted from Pearson product–moment coefficient or Spearman’s rank correlation coefficient. Significant trends are marked with regression lines.

We found no differences in the analyzed tracheid traits between the study sites (Table 1), both when considering tree rings representing the entire life span or only the outermost tree ring. Tracheid diameter frequency distribution calculated from the entire life span (Supplementary Figure 3) exhibited two peaks at both sites, representing latewood and earlywood tracheids. Tracheids between 2 and 4 μm, 4 and 6 μm, and 6 and 8 μm in diameter were more frequent on the limited site (*p* < 0.05), while those between 12 and 14 μm, 14 and 16 μm, and 16 and 18 μm in diameter were more frequent at the favorable site (*p* < 0.01). Tracheids wider than 48 μm only occurred in trees from the limited site. Considering the outermost tree ring, 6–8-μm tracheids were more frequent on the limited site (*p* = 0.047), while 10–12 μm, 12–14 μm, 14–16 μm, and 16–18 μm tracheids were more frequent on the favorable site (*p* < 0.05). Tracheids wider than 40 μm only occurred in trees on the limited site. In trees on the limited site, *d*_*h*_ increased toward the outer tree rings (Figure 2). On the favorable site, *d* decreased and *(t/b)*^2^ increased toward the outer tree rings.

**TABLE 1:** Comparison of analyzed tracheid traits, pit dimensions, and pit functional properties between study sites [*d* = mean tracheid diameter, *d*_*h*_ = mean hydraulic diameter, *(t/b)*^2^ = cell wall reinforcement, *D*_*p*_ = pit border diameter, *D*_*m*_ = margo diameter, *D*_*t*_ = torus diameter, *D*_*a*_ = aperture diameter, *F* = margo flexibility, *O*_*a*_ = absolute overlap, *O* = torus overlap, *V* = valve effect].

	All analyzed tree rings		Outermost tree ring	
	Limited site	Favorable site	Limited site	Favorable site
	**Tracheid traits**			

*d* (μm)	17.20 ± 0.9^a^	19.65 ± 1.0^a^	18.09 ± 1.2^a^	16.38 ± 1.1^a^
*d*_*h*_ (μm)	29.04 ± 1.1^a^	29.29 ± 1.1^a^	31.53 ± 1.5^a^	27.33 ± 1.9^a^
*(t/b)* ^2^	0.040 ± 0.004^a^	0.026 ± 0.004^a^	0.040 ± 0.007^a^	0.043 ± 0.011^a^

	**Pit dimensions**			

*D*_*p*_ (μm)	19.92 ± 0.26^a^	19.14 ± 0.33^a^	20.53 ± 0.44^a^	19.86 ± 0.50^a^
*D*_*m*_ (μm)	18.38 ± 0.26^a^	17.91 ± 0.30^a^	19.03 ± 0.43^a^	18.59 ± 0.49^a^
*D*_*t*_ (μm)	10.63 ± 0.20^a^	10.08 ± 0.20^a^	11.21 ± 0.32^a^	10.74 ± 0.31^a^
*D*_*a*_ (μm)	5.28 ± 0.10^a^	5.71 ± 0.12^b^	5.74 ± 0.16^a^	5.91 ± 0.21^a^

	**Pit functional properties**			

*F*	0.422 ± 0.005^a^	0.437 ± 0.003^b^	0.410 ± 0.006^a^	0.423 ± 0.006^a^
*O*_*a*_	2.05 ± 0.02^a^	1.78 ± 0.02^b^	1.95 ± 0.03^a^	1.83 ± 0.03^b^
*O*	0.503 ± 0.004^a^	0.433 ± 0.005^b^	0.519 ± 0.035^a^	0.447 ± 0.006^b^
*V*_*ef*_	0.212 ± 0.003^a^	0.188 ± 0.001^b^	0.211 ± 0.013^a^	0.188 ± 0.003^b^

*Different letters indicate significant differences (p < 0.05) between sites. Mean ± SE.*

### Pit Dimensions and Functional Properties

**FIGURE 3:**
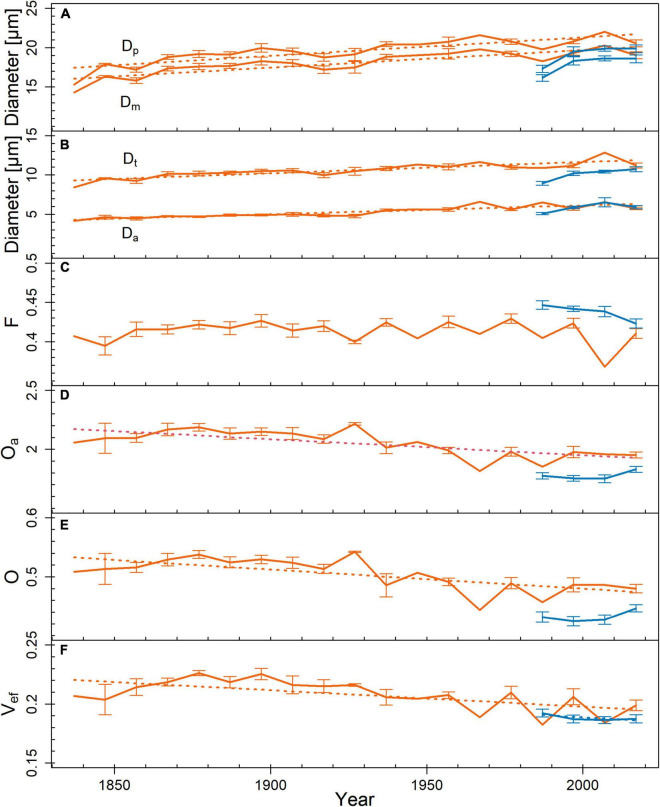
Trends in pit dimensions and functional properties across tree rings from pith to bark (red = limited site, blue = favorable site). **(A)** pit diameter (*D*_*p*_; *r*^2^_*limited*_ = 0.71, *p* < 0.001), margo diameter (*D*_*m*_; *r*^2^_*limited*_ = 0.72, *p* < 0.001), **(B)** torus diameter (*D*_*t*_; *r*^2^_*limited*_ = 0.74, *p* < 0.001), pit aperture diameter (*D*_*a*_; *r*^2^_*limited*_ = 0.85, *p* < 0.001), **(C)** margo flexibility (*F*), **(D)** absolute overlap (*O*_*a*_; *r*^2^_*limited*_ = 0.48, *p* < 0.001), **(E)** torus overlap (*O*; *r*^2^_*limited*_ = 0.48, *p* = 0.001), and **(F)** valve effect (*V*_*ef*_; *r*^2^_*limited*_ = 0.4, *p* = 0.004; *r*^2^_*favorable*_ = 0.54, *p* = 0.027). Mean ± SE. Measurement points without SE bars represent tree rings, which were sampled only in one tree individual. Given *r*^2^- and *p*-values resulted from Pearson product–moment coefficient or Spearman**’**s rank correlation coefficient. Significant trends are marked with regression lines.

**FIGURE 4:**
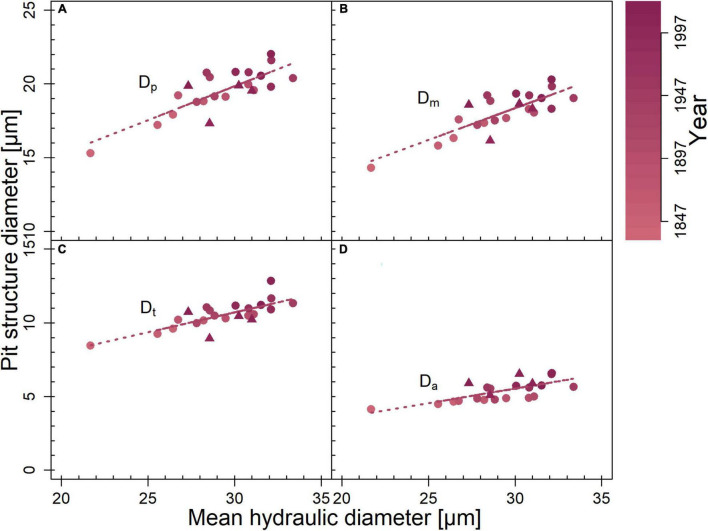
Correlation of mean hydraulic diameter with diameter of **(A)** pit border (*D*_*p*_; *r*^2^ = 0.8, *p* < 0.001), **(B)** margo (*D*_*m*_; *r*^2^ = 0.81, *p* < 0.001), **(C)** torus (*D*_*t*_; *r*^2^ = 0.77, *p* < 0.001), and **(D)** aperture (*D*_*a*_; *r*^2^ = 0.72, *p* < 0.001). Averaged per year for each site (circles = limited site, triangles = favorable site). Correlations were calculated for both sites together. Given *r*^2^- and *p*-values resulted from Pearson product–moment coefficient. Significant correlations are marked with regression lines. Color gradient indicates year.

Comparing pits from tree rings representing the entire life span, *D*_*a*_ was lower on the limited site than on the favorable site (*p* = 0.002; Table 1). In the outermost tree ring, pit dimensions were similar on both sites. Functional properties indicated several differences between the study sites: *F* was lower on the limited site than on the favorable site (*p* = 0.007). *O*_*a*_, *O*, and *V*_*ef*_ were higher on the limited than on the favorable site (*p* < 0.001). This was also observed in the outermost tree ring: *O*_*a*_, *O*, and *V*_*ef*_ differed (*p* < 0.05). On the limited site, *D*_*p*_, *D*_*m*_, *D*_*t*_, and *D*_*a*_ increased toward the outer tree rings (Figure 3). On the favorable site, no trends for the analyzed pit dimensions were observed from the inner to the outer tree rings, however, *D*_*t*_ tended to be larger in the outer tree rings. No trend was found in trees from the limited site for *F*, whereas *O*_*a*_, *O*, and *V*_*ef*_ were higher in the inner compared to the outer tree ring. On the favorable site, *F* tended to be and *V*_*ef*_ was lower in the outer than the inner tree rings. Furthermore, pit dimensions *D*_*p*_, *D*_*m*_, *D*_*t*_, and *D*_*a*_ scaled positively with *d*_*h*_ among the tree rings (Figure 4).

## Discussion

Tracheid traits and pit dimensions were similar in trees of both sites (in contrast to our hypothesis). However, at a similar distance to the treetop, pit functional properties *O*_*a*_, *O*, and *V*_*ef*_ were higher in trees from the limited site, indicating higher hydraulic safety. Furthermore, *d*_*h*_ and pit dimensions increased toward the outer tree rings in trees from the limited site, indicating higher hydraulic efficiency with increasing distance from the treetop.

### Slightly Higher Hydraulic Safety on the Limited Site

Although trees on the limited site were older than those on the favorable site, both sampled stands had similar tree heights because trees on the favorable site were growing faster (tree rings were on average 3.75 times wider; see also Supplementary Figure 2). The higher growth rate was most probably caused by higher nutrient and water availability due to different soil properties.

Despite the striking difference in radial growth between the sites, tracheid dimensions were similar (Table 1). Accordingly, Kiorapostolou et al. (2018) found no difference in conduit lumen area in *P. sylvestris* saplings exposed to different water availability and sampled at the same distance from the treetop. Generally, the allometric scaling with distance from the treetop (or branch tip) seems to be the main driver of conduit diameters, whereas environmental conditions have low influence (Olson et al., 2018, 2021). Deviating from the optimal scaling pattern may decrease a plant’s fitness by pushing hydraulic efficiency and hydraulic safety out of balance (Olson et al., 2021). For example, Kiorapostolou et al. (2020) found larger tracheid diameters in *P. sylvestris* trees declining from drought stress than in non-declining trees at the same distance from the treetop.

In addition to conduits, pits are crucial for hydraulic efficiency and safety. Pit functional properties *O*_*a*_, *O*, and *V*_*ef*_, which were higher in trees on the limited study site compared to those on the favorable site (Table 1), indicated slightly higher hydraulic safety, as these properties play a crucial role in various air-seeding mechanisms (Domec et al., 2008; Delzon et al., 2010). Seal capillary seeding, occurring when the torus–aperture seal is insufficiently airtight, is the most probable air-seeding mechanism in *P. sylvestris* (Bouche et al., 2014), possibly due to the uneven surface of the pit border (Figure 4). A larger torus relative to the pit aperture (i.e., *O*_*a*_ or *O*) increases resistance against air seeding by this mechanism (Delzon et al., 2010). However, with increasing torus diameter, hydraulic efficiency decreases, which Roskilly et al. (2019) suggested to be contributing to the trade-off between longevity and fast growth in conifers.

Pit dimensions change with tracheid size within tree rings, and even small variations in pit dimensions affect the tracheid hydraulic functions (Sviderskaya et al., 2021). Our selection of randomly cut open pits, which mainly occurred in large earlywood conduits, may have led to a small overestimation of pit dimensions in tree rings with a higher proportion with large conduits. However, the potential bias, particularly concerning the pit functional properties, is small, as the proportions of large earlywood tracheids were similar on both sites (Supplementary Figure 3).

### Increasing Hydraulic Efficiency From Inner to Outer Tree Rings

**FIGURE 5:**
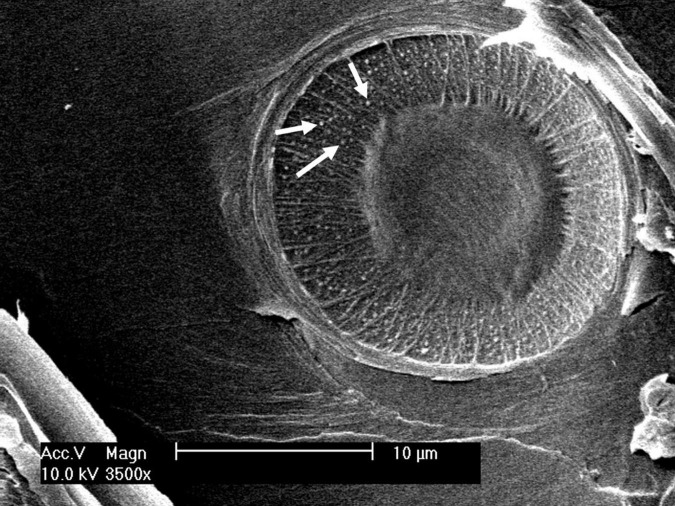
Uneven surface structure (white arrows) of a pit border visible through an aspirated margo.

In trees from the limited site, *d*_*h*_ and pit dimensions increased from the inner to the outer tree rings (Figures 2, 5), leading to increased hydraulic efficiency at breast height as the trees grew. Trees, therefore, adjusted their tracheids and pits to the increasing distance from the treetop to counteract the increasing pathway length resistance (compare Lintunen and Kalliokoski, 2010; Anfodillo et al., 2013; Lazzarin et al., 2016; Losso et al., 2018; Christof et al., 2020). Existing deviation from the linear regression may on the one hand result from unknown height growth dynamics of the tree individuals. On the other hand, it may indicate, that other factors besides the distance from the treetop may have some influence on tracheid and pit dimensions. Castagneri et al. (2015) found the mean tracheid lumen area in *P. abies* to be sensitive to summer precipitation and suggested that water availability could influence tracheid enlargement.

Simultaneously with increasing pit dimensions, functional properties *O*_*a*_, *O*, and *V*_*ef*_ decreased toward the outer tree rings (Figure 2), which indicates a trade-off between hydraulic efficiency and safety at the pit level (Domec et al., 2006). However, as trees grow, they develop an increasingly efficient root system (Brodribb et al., 2010), and the improving water supply may allow a slightly decreasing hydraulic safety in favor of increasing hydraulic efficiency at breast height. On the contrary, Domec et al. (2008) and Losso et al. (2018) found *O*_*a*_ to increase with tree height in *Pseudotsuga menziesii*, *P. abies*, and *Pinus cembra*.

Trees on the favorable site showed a decreasing trend from the inner to the outer tree rings in *V*_*ef*_, similar to the trees from the limited site (Figure 2). For the short time span of 37 years, the sampling interval of every 10th tree ring was possibly insufficient to show trends in *d*_*h*_, pit dimensions, and functional properties. The deviation due to height growth dynamics or possibly due to external influences (as mentioned above for the trees from the limited site) may blur the trends. According to Anfodillo et al. (2013) and Kiorapostolou et al. (2020), the adjustment of hydraulic efficiency to transport distance (i.e., distance from the treetop) is independent of environmental conditions. An increasing proportion of latewood in the tree rings due to the opposite trends of earlywood width and latewood width (Supplementary Figure 2) explains the negative trend in *d* toward the outer tree rings.

Because pits can contribute ≥ 50% to the xylem’s total hydraulic resistance (Domec et al., 2006; Choat et al., 2008), trees under any condition need to coordinate *d*_*h*_ and pit dimensions (Figure 3; Hacke et al., 2004; Lazzarin et al., 2016) to optimally adjust hydraulic efficiency.

## Conclusion

Despite the contrasting growth rates due to different environmental conditions, *P. sylvestris* trees of similar height formed similar-sized tracheids. Only the pit architecture differed, indicating slightly higher hydraulic safety on the limited site and higher efficiency on the favorable site. This suggests that maintaining efficient xylem transport is necessary for tree survival under any condition, whereas the importance of hydraulic safety increases under stressful conditions. The distinctly older trees on the limited site adjusted their *d*_*h*_ and pit dimensions during their lifetime according to the growing distance from the treetop and clearly coordinated these traits with each other to increase their hydraulic efficiency at breast height. Our results provide evidence that under contrasting growing conditions, intra-specific variation in tracheid and pit traits is mainly driven by the distance from the treetop. However, even at a similar distance from the treetop, small but significant variations in pit traits occur, which may affect tree hydraulic safety and efficiency under drought.

## Data Availability Statement

The raw data supporting the conclusions of this article will be made available by the authors, without undue reservation.

## Author Contributions

MH conducted the practical work, analyzed the data, prepared the figures, and wrote the text. SM and WO designed the study and supervised the work. AG outlined the figure concept. SM, WO, AG, and AL revised and commented on the text and figures. All authors contributed to the article and approved the submitted version.

## Conflict of Interest

The authors declare that the research was conducted in the absence of any commercial or financial relationships that could be construed as a potential conflict of interest.

## Publisher’s Note

All claims expressed in this article are solely those of the authors and do not necessarily represent those of their affiliated organizations, or those of the publisher, the editors and the reviewers. Any product that may be evaluated in this article, or claim that may be made by its manufacturer, is not guaranteed or endorsed by the publisher.
