# Supplementary material for: Tracheid and Pit Dimensions Hardly Vary in the Xylem of Pinus sylvestris Under Contrasting Growing Conditions
Source: Front Plant Sci. 2021 Dec 21;12:786593. doi: 10.3389/fpls.2021.786593 (PMC8725801; doi:10.3389/fpls.2021.786593)
Supplement: Supplementary file 1 [file Data_Sheet_1.docx]

***Supplementary Material***

|  | **Supplementary table 1**. Study design. Selected tree rings (TR) for tracheid analysis (TA) and pit analysis (PA) are marked with “x”. Those marked with “(x)” were included in trend analysis but excluded from site comparison. Limited site: trees 1-10, favorable site: trees 11-20. |
| --- | --- |


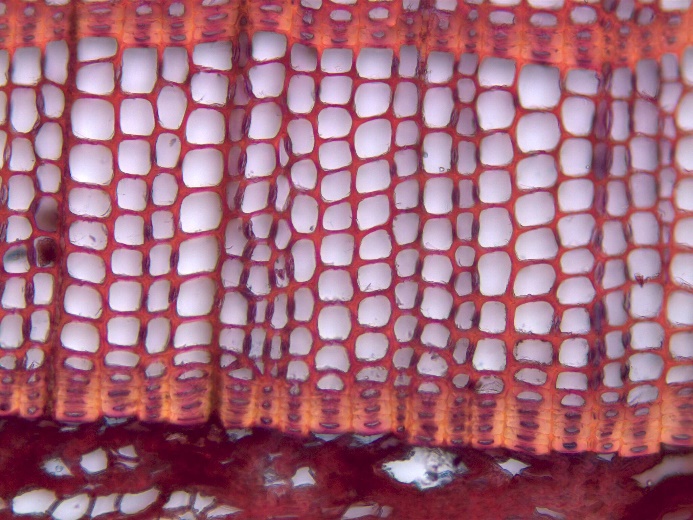

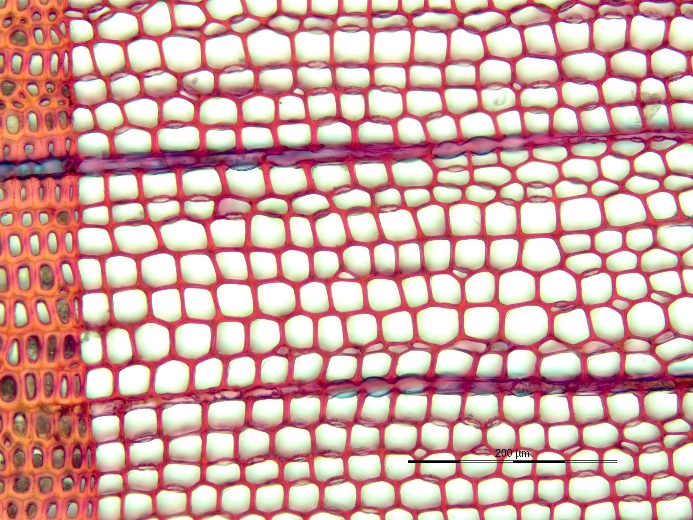

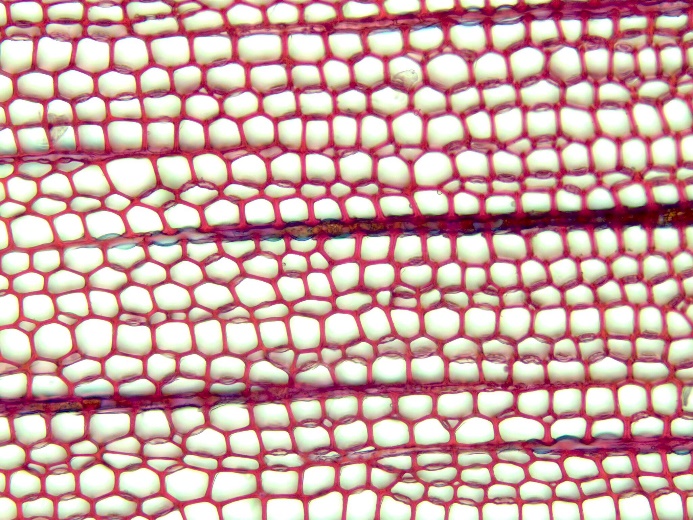

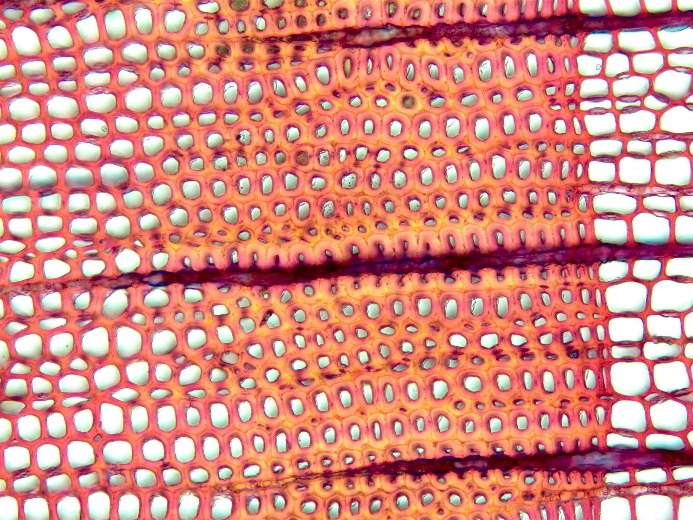

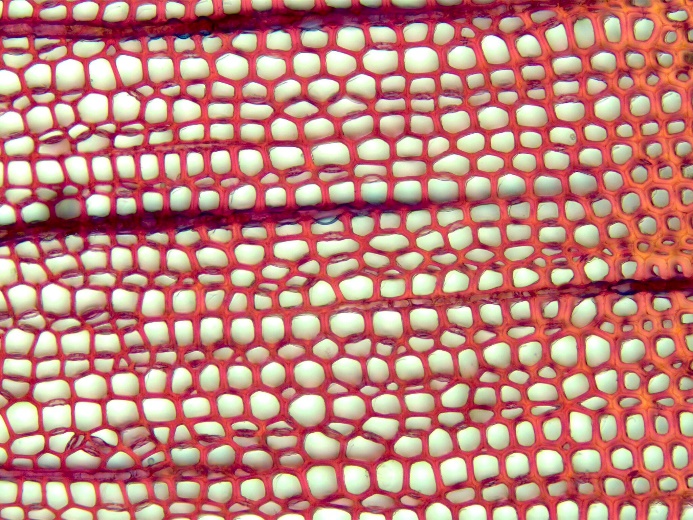

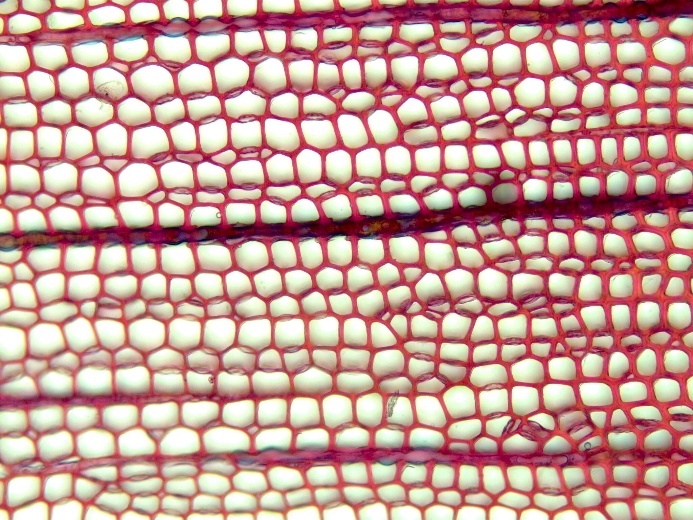


Direction of growth

Direction of growth

E

C

F

D

B

A

**Supplementary Figure 1.** Example images of stained cross sections prepared from tree cores as used for tracheid analysis showing (A) a tree ring of a tree from the limited site and (B-F; images overlapping in the direction of growth) from the favorable site. The scale bar in (B) applies to all images (A-F).

| 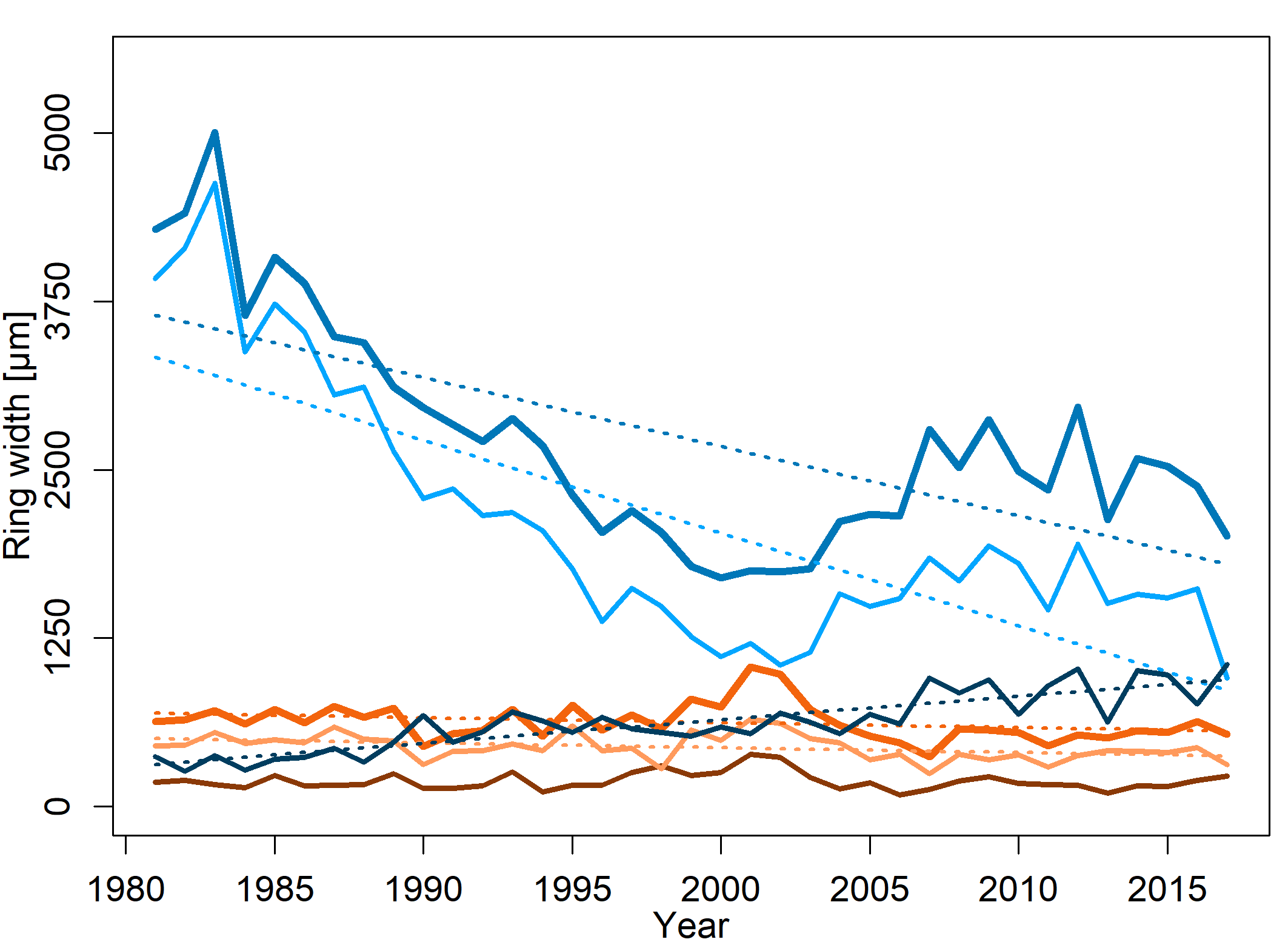 |
| --- |
| **Supplementary Figure 2**. Ring-width chronologies (1981-2017). Limited site: total tree-ring width (orange, r² = 0.05, p = 0.002), earlywood width (light orange, r² = 0.07, p < 0.001) and latewood width (brown). Favorable site: total tree-ring width (blue, r² = 0.35, p < 0.001), earlywood width (light blue, r²= 0.5, p < 0.001), latewood width (dark blue, r² = 0.74, p < 0.001). Significant trends are marked with regression lines. |

| 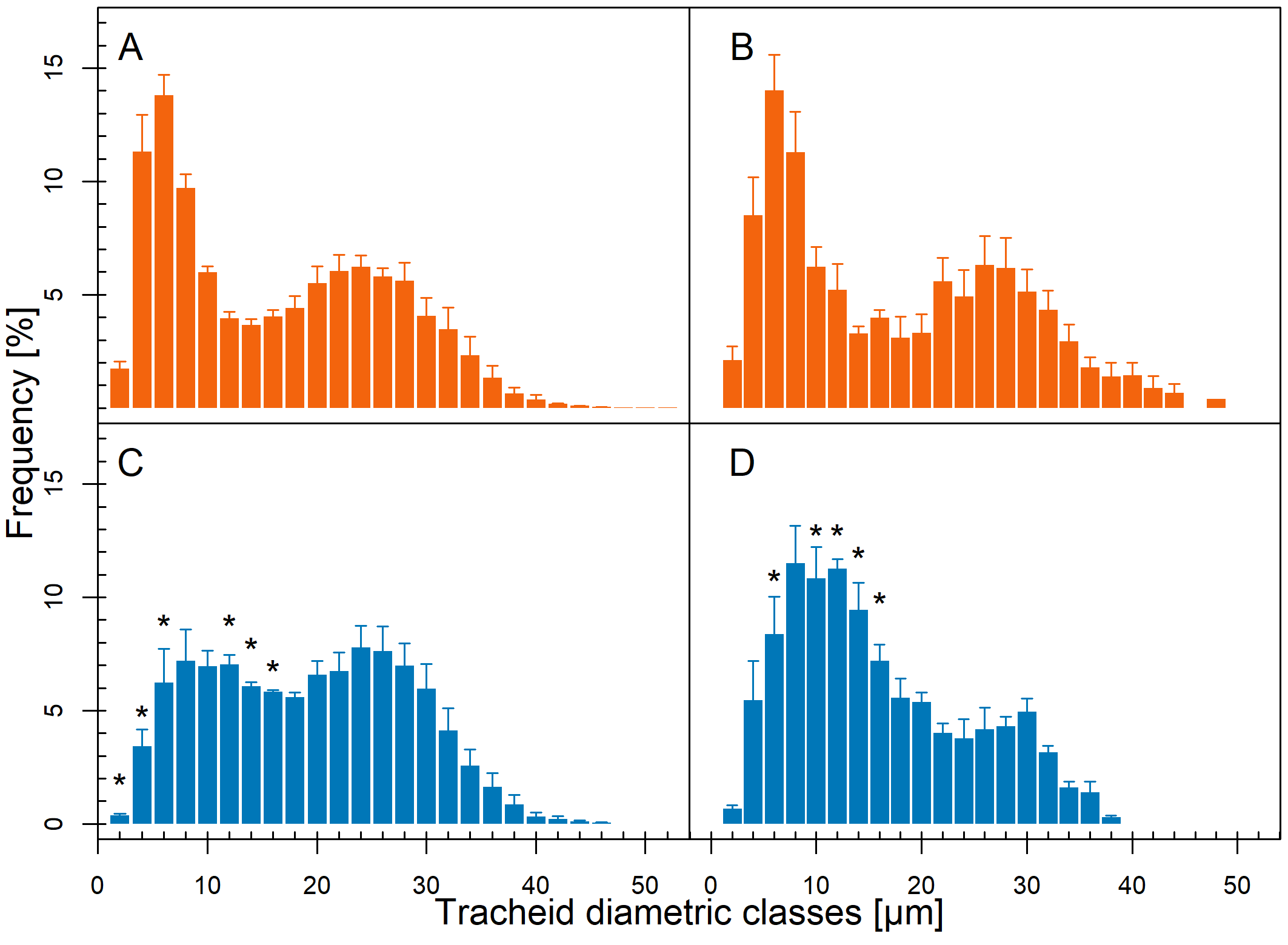 |
| --- |
| **Supplementary Figure 3**. Frequency distribution of tracheid diametric classes (2-µm classes) in all analyzed tree rings on limited (A) and favorable site (B) and in the outermost tree ring on limited (C) and favorable site (D). Mean ± SE. Significant differences (p < 0.05) between sites are marked with stars in c and d. |
