# Supplementary figures and images for: Tracheid and Pit Dimensions Hardly Vary in the Xylem of Pinus sylvestris Under Contrasting Growing Conditions
Source: Front Plant Sci. 2021 Dec 21;12:786593. doi: 10.3389/fpls.2021.786593 (PMC8725801; doi:10.3389/fpls.2021.786593)

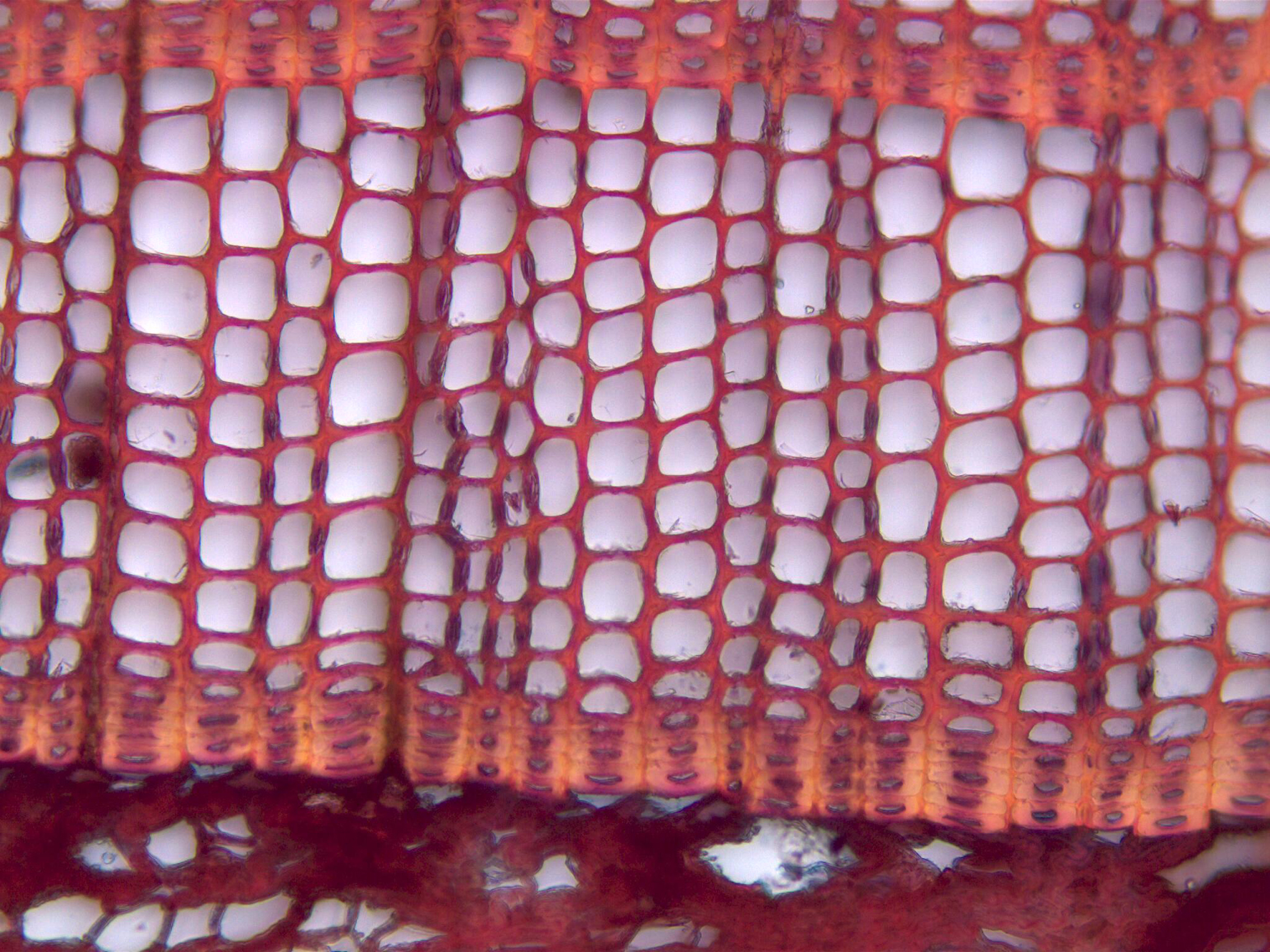

Supplement: Supplementary file 2 [file Image_1.JPEG]

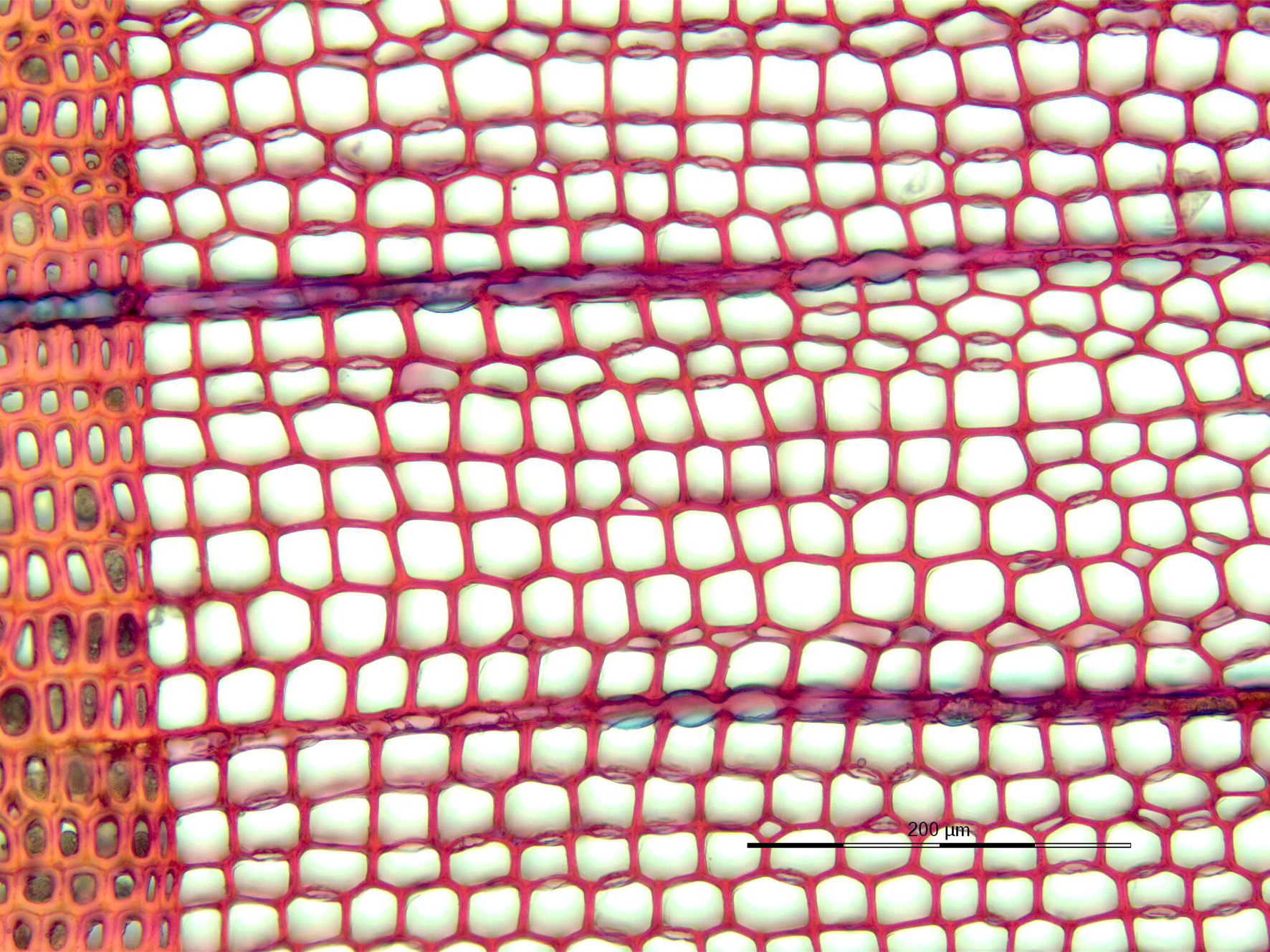

Supplement: Supplementary file 3 [file Image_2.JPEG]

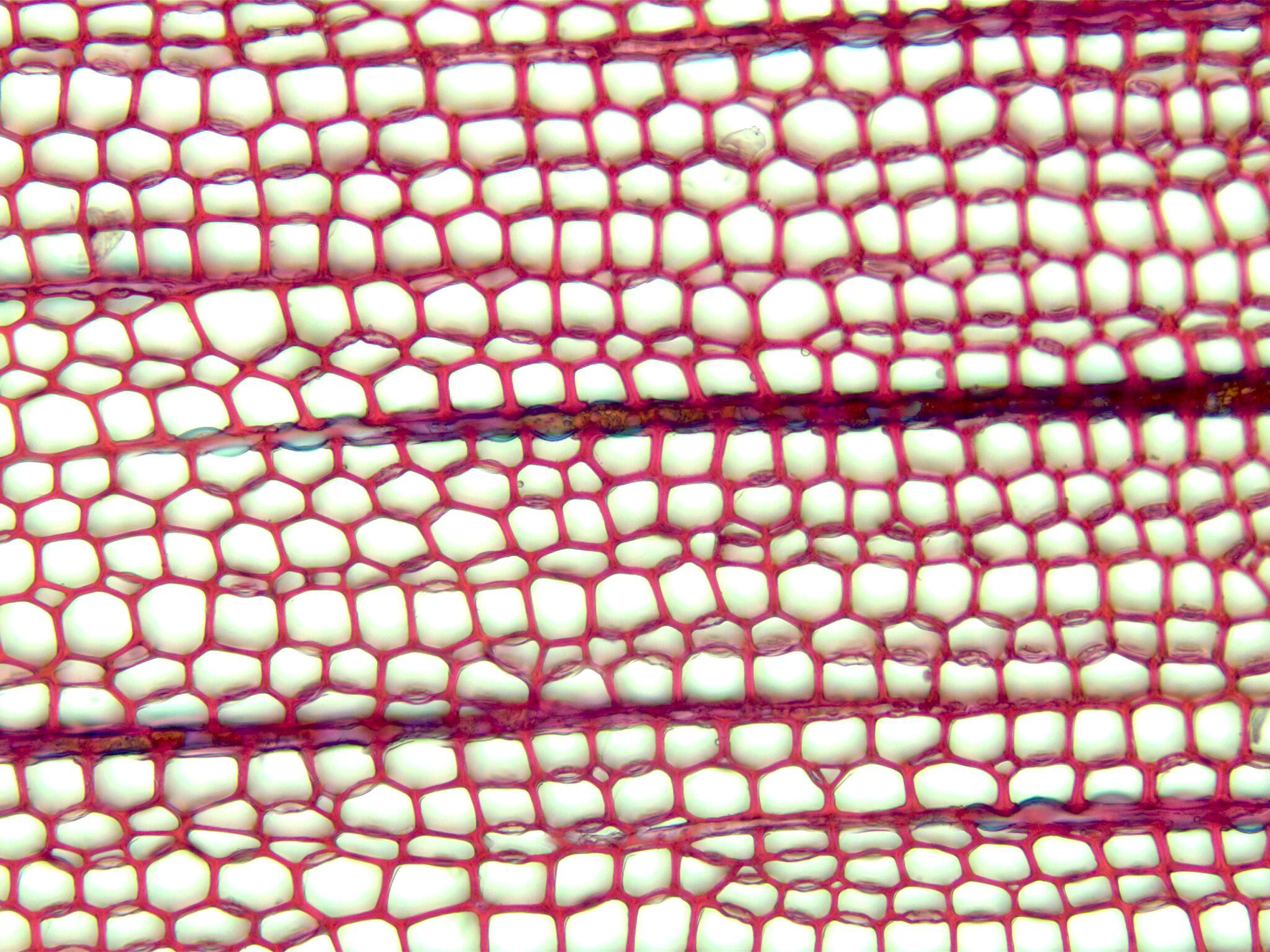

Supplement: Supplementary file 4 [file Image_3.JPEG]

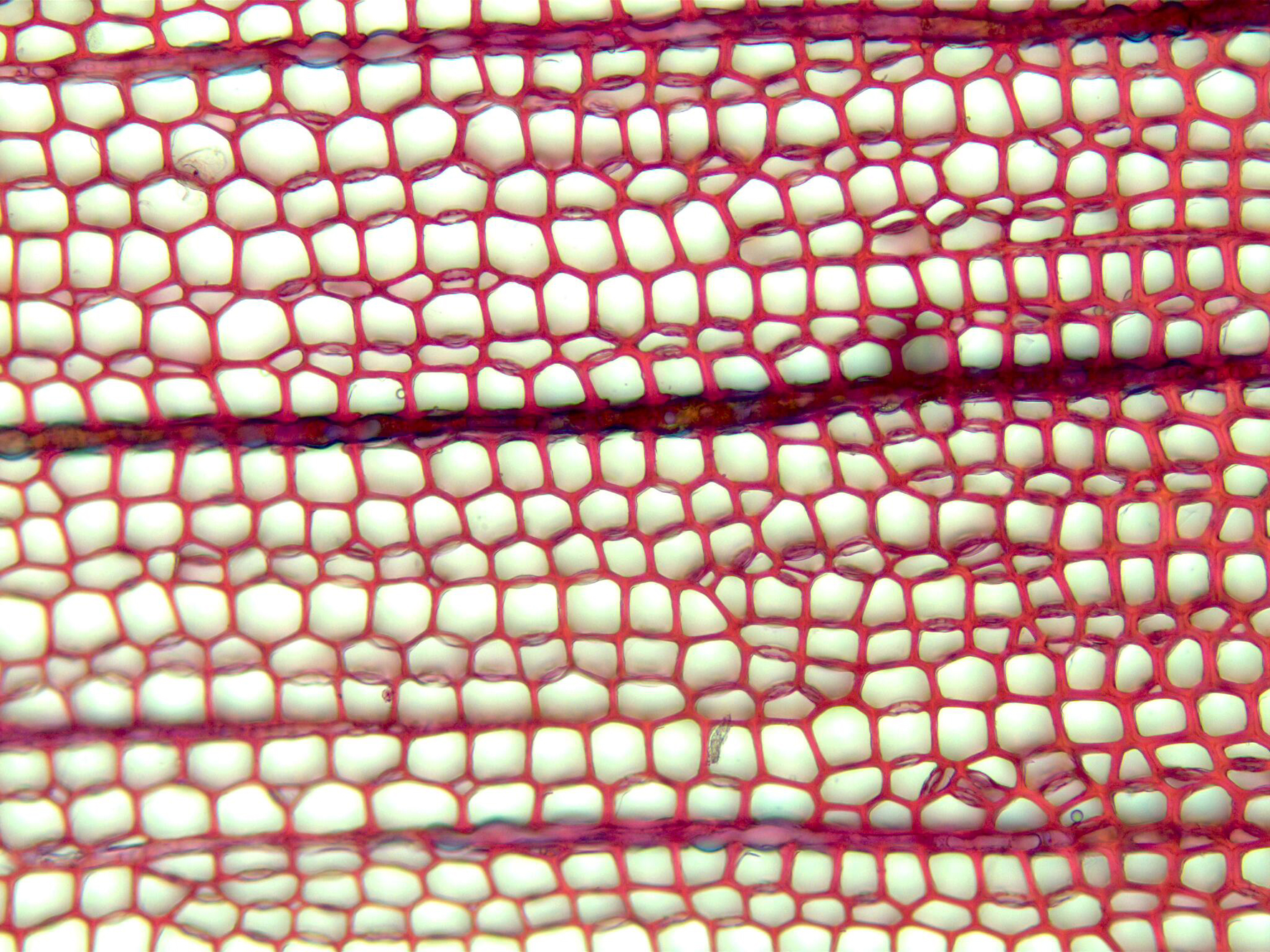

Supplement: Supplementary file 5 [file Image_4.JPEG]

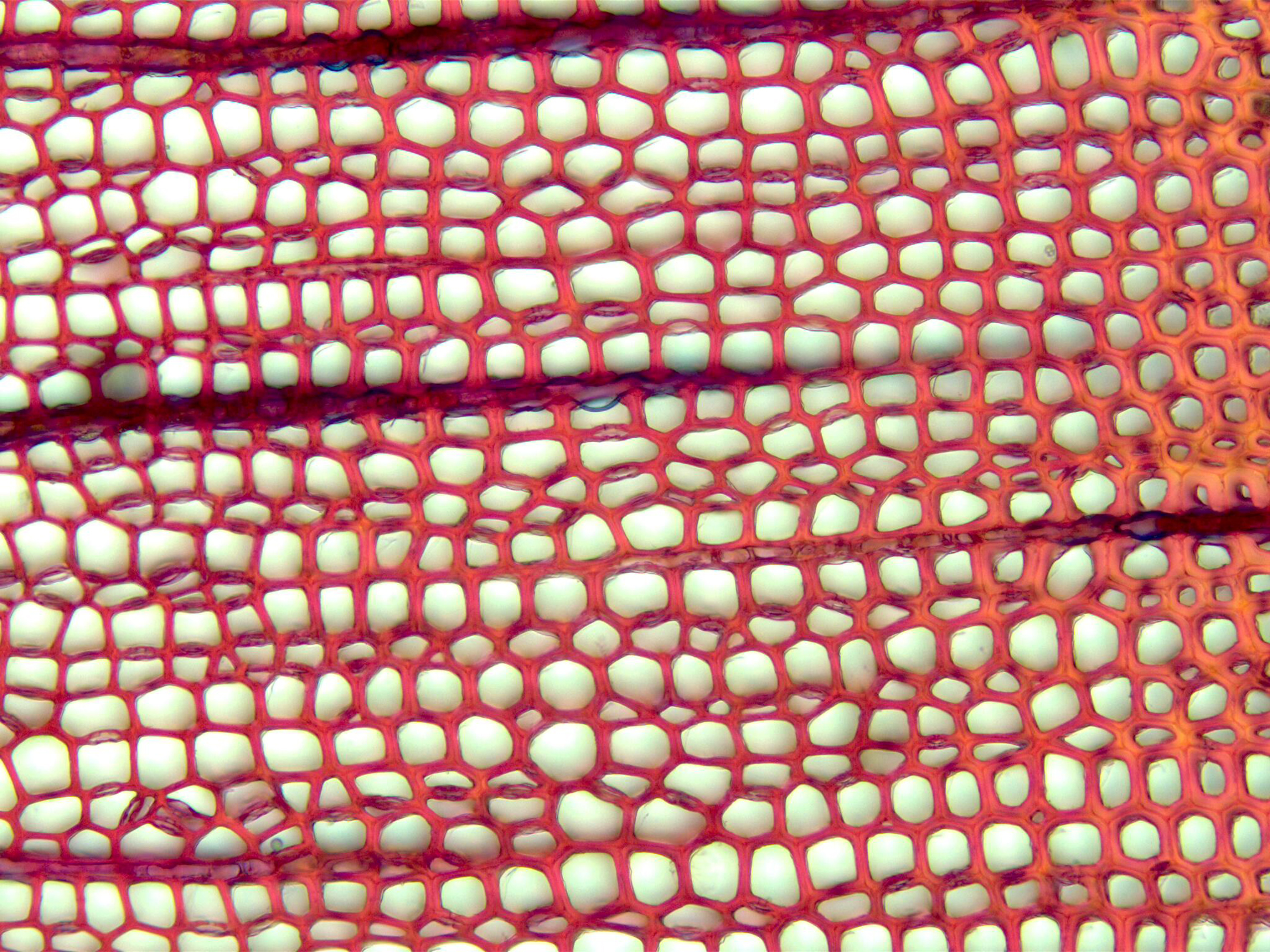

Supplement: Supplementary file 6 [file Image_5.JPEG]

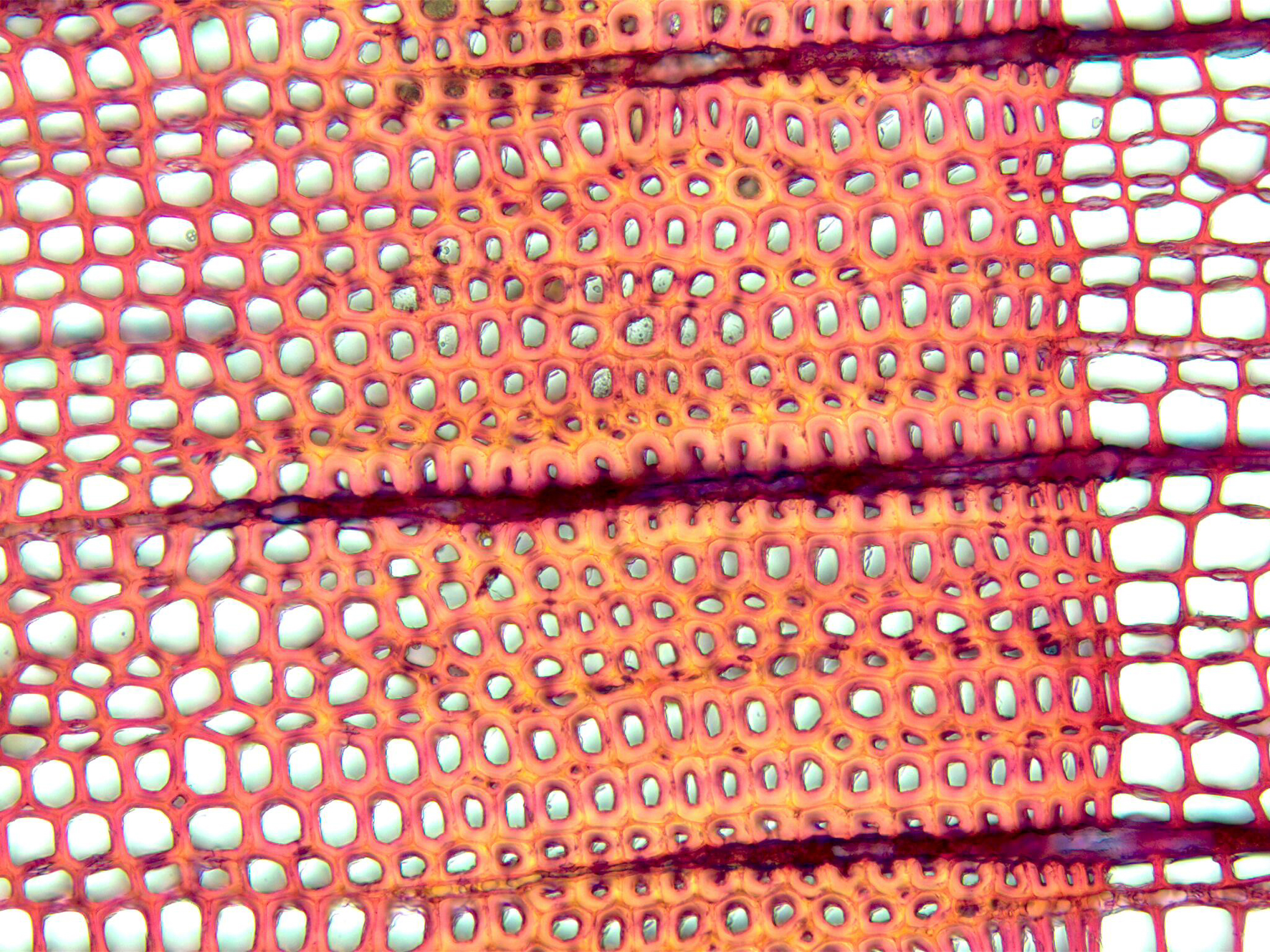

Supplement: Supplementary file 7 [file Image_6.JPEG]
